# Supplementary material for: Toughness and strength of nanocrystalline graphene
Source: Nat Commun. 2016 Jan 28;7:10546. doi: 10.1038/ncomms10546 (PMC4738364; doi:10.1038/ncomms10546)
Supplement: Supplementary Information — Supplementary Figures 1-5, Supplementary Notes 1-3 and Supplementary References. [file ncomms10546-s1.pdf]

Supplementary Figures

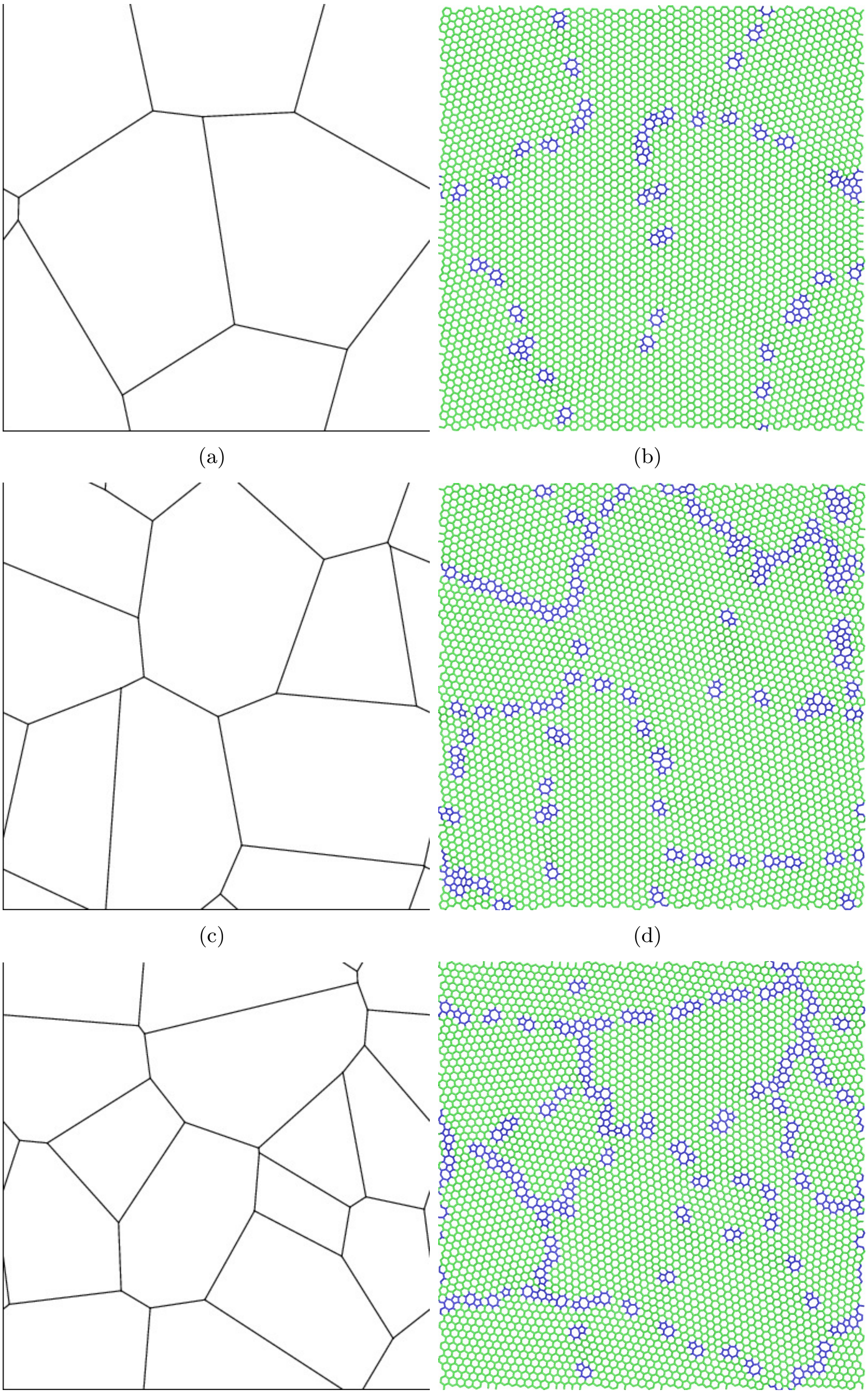

(a) (b)

(c) (d)

(e) (f)

Supplementary Figure 1: **Numerically generated nanocrystalline graphene.** 2D periodic nanocrystalline sample of size 128 Å with 4, 8, and 12 grains, respectively.

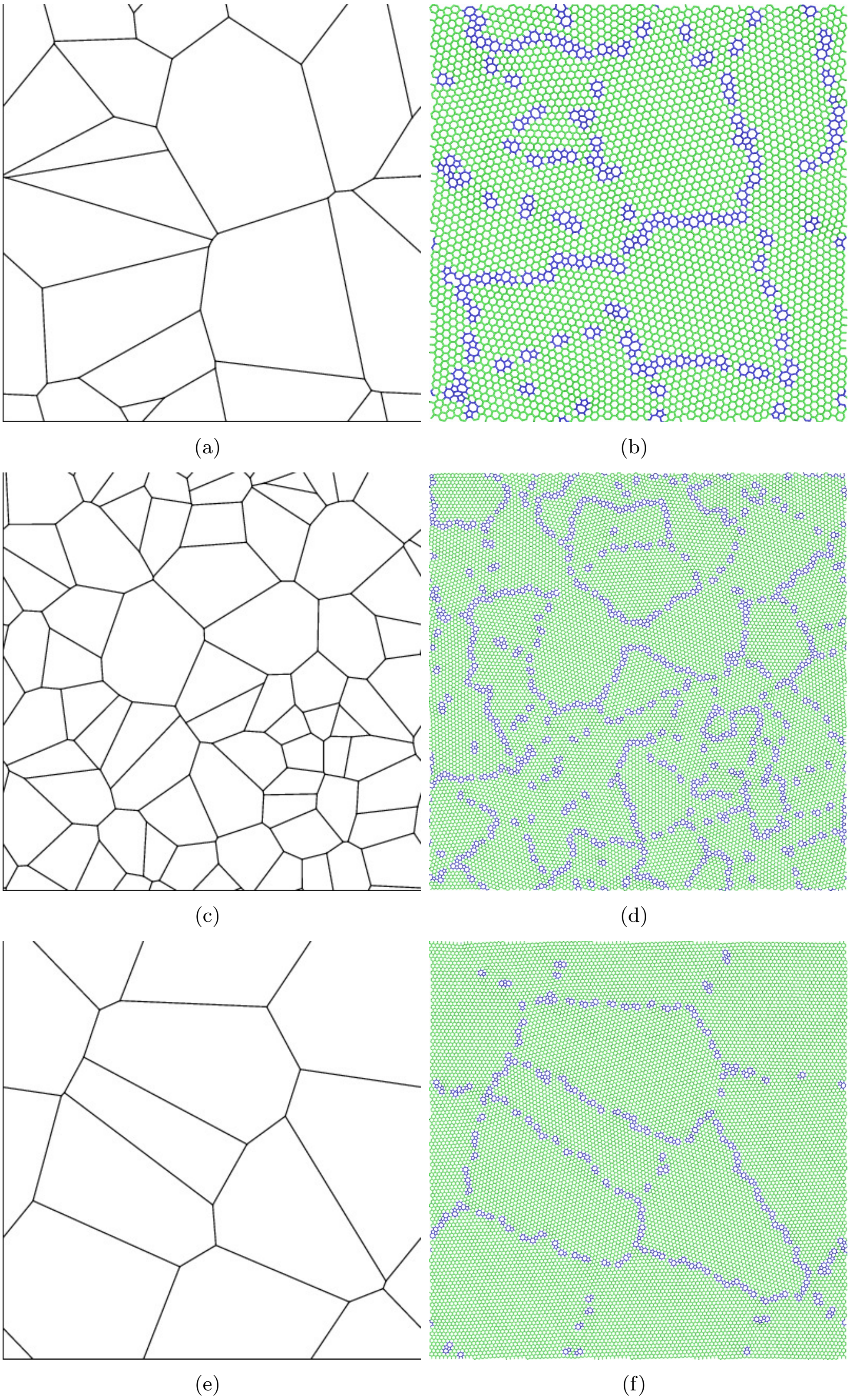

Supplementary Figure 2: **Numerically generated nanocrystalline graphene.** 2D periodic nanocrystalline samples.(a) Size 128 Å, 16 grains, (b) size 256 Å, 64 grains, and (c) size 256 Å, 8 grains.

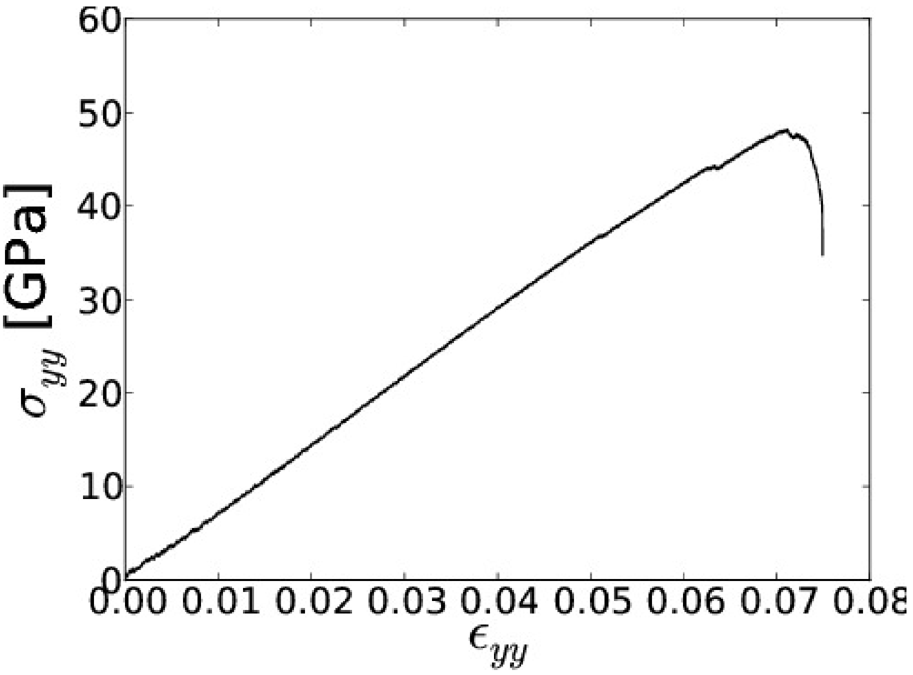

Supplementary Figure 3: **Elastic response of nanocrystalline graphene.** The stress-strain response of a 2D periodic nanocrystalline samples with grain size 64 Å, loaded at a strain rate of $\dot{\epsilon}_{yy} = 10^9 \mathrm{s}^{-1}$ at $T = 300$K. ϵ_{yy} is the applied strain in the y-direction, while σ_{yy} is the measured stress in the y-direction.

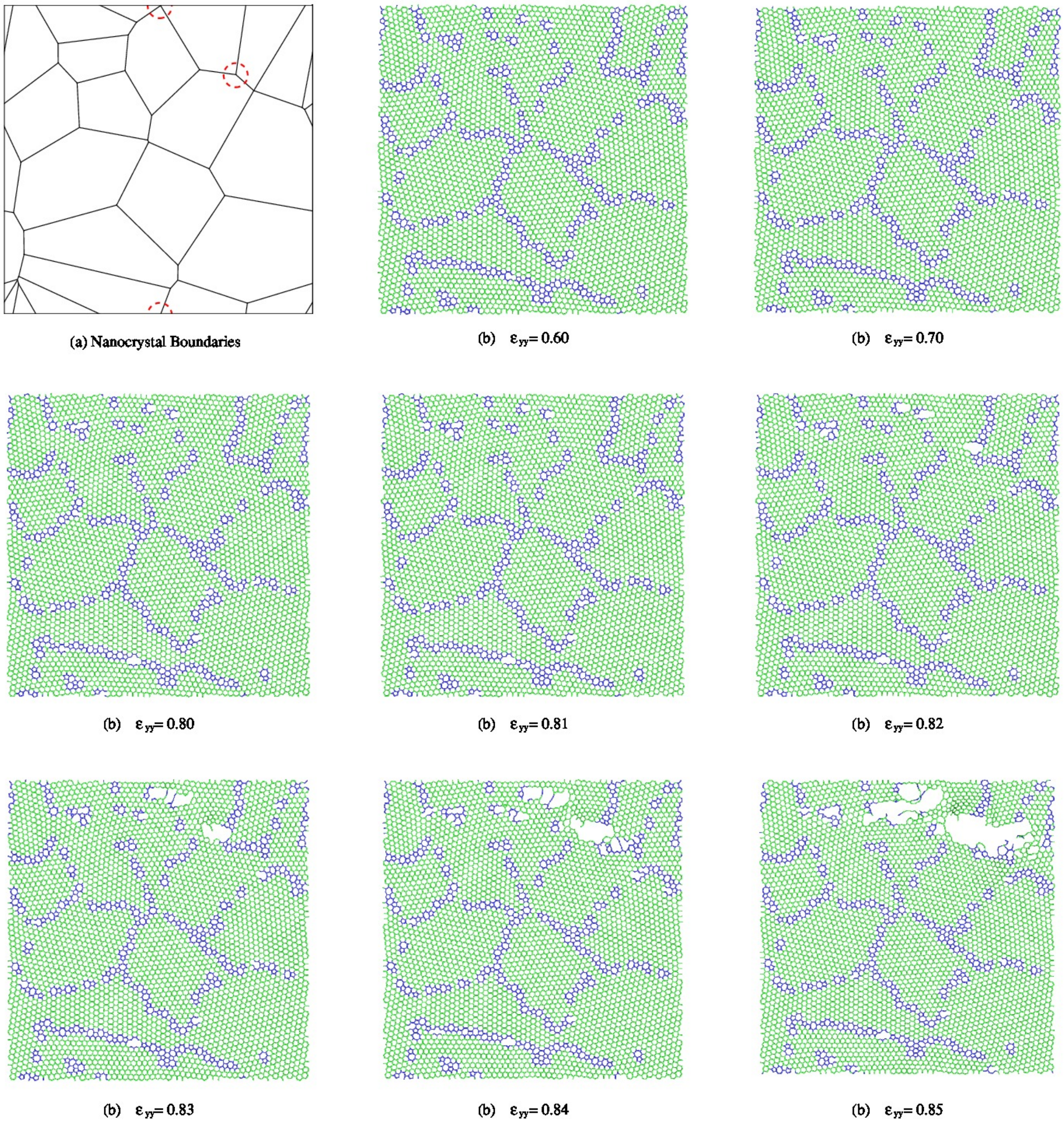

Supplementary Figure 4: **Failure of polycrystalline graphene under tension.** Snapshots of simulation of uniaxial straining of a 2D periodic nanocrystalline sample.(a) Nanocrystalline domain boundaries, size 128 Å, 16 grains. The triple junctions near which the fatal flaws eventually develop are marked by the dashed red circles. (b)-(i) Simulation snapshots at various levels for strain ϵ_{yy} (the applied strain in the y-direction). Complete failure occurs soon after $\epsilon_{yy} = 0.85$.

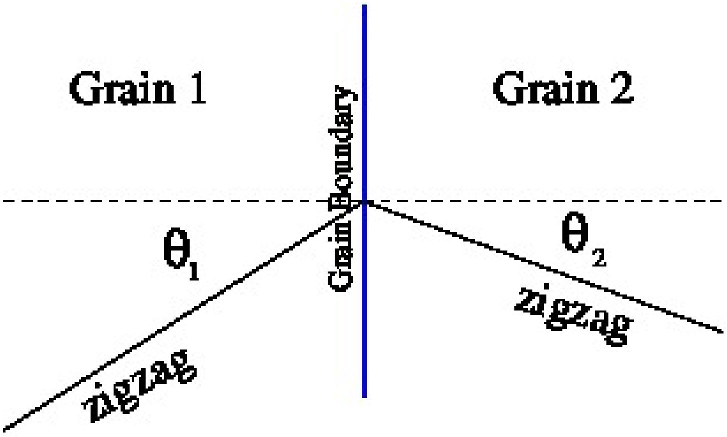

(a)

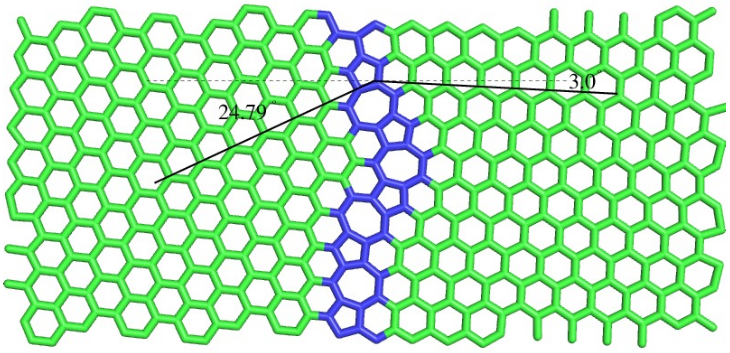

(b)

Supplementary Figure 5: **Illustration of GB geometry in graphene.** (a) Geometry of a general grain boundary in graphene. (b) An example with $\theta_1 = 24.79°$, $\theta_2 = 3.0°$.

Supplementary Note 1. Simulations

Creating nanocrystalline graphene sheets for simulation

We use the following method to generate nanocrystalline graphene sheets with random grain random shapes and orientations. A square sheet of size L and grain size μ has $n_g = L^2/\mu^2$ grains. First we choose the n_g points as 'centers' of the grains at random (distributed uniformly on the sheet of area L^2). Then a Voronoi construction with these points is used to generate the granular regions associated with them. Then the orientation of lattice vectors in each grain is chosen at random. The positions of carbon atoms in the grains and at the grain boundaries is assigned with a recently proposed algorithm[1]. This algorithm results in well annealed nanocrystals. Supplementary Fig. 1 (a), (c), (e) show some examples of nanocrystalline domains generated with this method. Supplementary Fig. 1 (b), (d), (f) show the corresponding atomic positions. Supplementary Fig. 2 shows some more nanocrystalline morphologies.

Simulations of nanocrystalline strength

We simulate 24 different combinations of (L, μ, $\dot{\epsilon}$) (64, 32, 1), (128, 64, 1), (128, $32\sqrt{2}$, 1), (128, $64/\sqrt{3}$, 1), (128, 32, 1), (256, 128, 1), (256, $64\sqrt{2}$, 1), (256, 64, 1), (256, $32\sqrt{2}$, 1), (256, $256/5\sqrt{2}$, 1), (256, 32, 1), (512, 32, 1), (128, $32\sqrt{2}$, 0.5), (128, $64/\sqrt{3}$, 0.5), (256, $64\sqrt{2}$, 0.5), (256, $32\sqrt{2}$, 0.5), (256, $256/5\sqrt{2}$, 0.5), (256, 32, 0.5), (128, $32\sqrt{2}$, 0.25), (128, $64/\sqrt{3}$, 0.25), (256, $64\sqrt{2}$, 0.25), (256, $32\sqrt{2}$, 0.25), (256, $256/5\sqrt{2}$, 0.25), (256, 32, 0.25), where L is the side of the square nanocrystalline graphene sheet, μ is the grain size, and $\dot{\epsilon}$ is the strain rate. The units for L, μ are Å, and the units for $\dot{\epsilon}$ are 10^9s^{-1}. For each combination of parameters we perform statistical sampling by simulating 10^4, 10^3, 10^2, 10^2 random nanocrystals for L = 64, 128, 256, 512 Å, respectively. Thus, for the simulations of strength we simulate a total of 19,500 nanocrystals. The simulations are carried out in the canonical ensemble with constant NVT integration using a Nose/Hoover thermostat with the LAMMPS software[2]. A constant strain rate is imposed in the y direction by using the SLLOD equations of motion[3]. The temperature is set to 300 K. The interaction between the carbon atoms is modeled by using the AIREBO potential[4–6], with the modification to the interaction cutoff parameter rc_{min} applied as suggested in Ref. 6. As the applied strain increase, the stress also increases initially. However, eventually fracture is initiated and the sample fails. The peak stress obtained during the loading process is defined as the strength of the nanocrystal. The stress-strain response of a typical polycrystalline sample

loaded uniaxially is shown in Supplementary Fig. 3. The thermal component of the stress is subtracted from the net response. Supplementary Fig. 4 shows the loading of a representative nanocrystal in this manner.

Simulations of nanocrystalline toughness

We evaluated the toughness of 500 samples of nanocrystalline graphene each for grain sizes of $\mu = 16, 32, 64$ Å. Nanocrystalline toughness is evaluated by generating a square nanocrystalline graphene sheet of size $L = 256$ Å with the required grain size and random grain morphology as discussed in the previous sections. A crack tip is introduced at the center of the nanocrystal by applying the deformation field corresponding to a stress intensity factor K_I as calculated from linear elastic fracture mechanics, i.e.,

$$u_x = \frac{K_I}{2G}\sqrt{\frac{r}{2\pi}}(\kappa - \cos\theta)\cos\theta/2, \quad u_y = \frac{K_I}{2G}\sqrt{\frac{r}{2\pi}}(\kappa - \cos\theta)\sin\theta/2, \tag{1}$$

where r, θ are the polar coordinates of a point with the origin placed at the crack tip, G is the shear modulus, and $\kappa = (3-\eta)/(1+\eta)$, and η is the Poisson's ratio. Atoms outside a radius of 100 Å from the crack tip are held fixed at this displacement, while atoms inside this radius are evolved with NVT dynamics. As before, we use the AIREBO potential to model the carbon-carbon interaction. The stress intensity factor K_I is incremented in steps of 0.1 MPa$\cdot$m$^{1/2}$, and the system is held at each K_I for 1 ps. The critical stress intensity factor, and thus the fracture toughness, is defined as the lowest value of K_I for which the crack grows in this manner.

Simulation of strength of graphene grain boundaries

Grain boundaries (GBs) in graphene can be parameterized with two angles, θ_1, θ_2 as shown in Supplementary Fig. 5 [7]. Another widely used parameterization employs the misorientation angle $\theta_M = \theta_1 + \theta_2$, and the line angle $\theta_L = |\theta_1 - \theta_2|$, which is entirely equivalent to the parameterization used here. The 6-fold rotation symmetry of the graphene lattice means $0 \leq \theta_1, \theta_2 < \pi/3$. The reflection symmetry about the grain boundary results in the symmetry operation $(\theta_1,\ \theta_2) \rightarrow (\theta_2,\ \theta_1)$, thus reducing the space of unique boundaries to $\theta_1 \geq \theta_2$. The mirror symmetry about the horizontal axis leads to the operation $(\theta_1, \theta_2) \rightarrow (\pi/3 - \theta_2, \pi/3 - \theta_1)$, thus further requiring that $\theta_1 + \theta_2 < \pi/3$. Thus, the space of unique grain boundaries is reduced to a triangle in the $(\theta_1,\ \theta_2)$ space.

In a recent publication[1], we grid this space in steps of 0.5° and generate a grain boundary at each point. We generate GBs with a width of 50 Å. We simulate fracture of the grain boundaries under uniform strain applied perpendicular to the grain boundary. The simulation method is similar to that used for nanocrystalline strength, the difference being that the GBs are periodic only along the GB direction. Thus, to apply a strain loading, a strip of atoms 5 Å wide at the left and right edges of the GB is moved rigidly at the required strain rate. The stress is recorded, an the strength of the GB is defined as the largest stress achieved during the simulation. Since we used a uniform grid, and random grain boundaries sample from a uniform probability density over the $(\theta_1,\ \theta_2)$ space, we can readily obtain an approximation for the survival probability, $\hat{S}_{GB}(\sigma)$, of a randomly chosen grain boundary. This probability is simply equal to the number of GBs that survived at the stress σ divided by the total number of simulated GBs. A plot of this survival probability can be found in Figure 5 of the main text.

Supplementary Note 2. Theoretical derivations

Detailed derivation of strain-rate and grain-size dependent survival probability of nanocrystalline graphene

Consider an isolated defect in the graphene sheet which has a stress dependent energy barrier given by $\Delta E(\sigma(t))$ associated with it, where $\sigma(t)$ is the stress at time t. In the case of polycrystalline graphene being considered here, this defect corresponds to individual pentagon-heptagon pairs that make the graphene GBs and TJs as shown in Figures 1b and 2b of the main text. If this barrier can be overcome by thermal fluctuations, then the defect will grow and cause global failure. According to nucleation theory, the probability that the barrier will be overcome in a small time dt is given by $\omega e^{-\Delta E(\sigma(t))/kT} dt$, where ω is a prefactor. Thus, the probability that the defect survives during the time dt is given by:

$$S_0(\sigma(t), dt) = 1 - \omega e^{-\Delta E(\sigma(t))/kT} dt \sim \exp\left(-\omega e^{-\Delta E(\sigma(t))/kT} dt\right). \tag{2}$$

Since $\sigma(t) = Y_R \dot{\epsilon} t$, where $Y_R = Y/(1-\eta^2)$ is the reduced modulus of elasticity for plane stress, Y is the Young's modulus, and η is the Poisson's ration, we can remove the explicit dependence on time from the above equation and get the probability that the defect survives a stress increment

of $d\sigma = Y_R\dot{\epsilon}dt$ in time increment dt as:

$$S_0(d\sigma) = \exp\left(-(\omega/Y_R\dot{\epsilon})e^{-\Delta E(\sigma)/kT}d\sigma\right). \quad (3)$$

Now consider a population of non-interacting defects with a stress dependent density of barrier heights given by $f(\Delta E(\sigma))$, and an area-density given by ρ. There are $\rho L^2 f(\Delta E)d\Delta E$ defects with barrier height ΔE in a sample of area L^2. The probability that all of the defects with barrier height ΔE survive the stress increment of $d\sigma$, which happens in time increment dt is simply a product of the individual probabilities, and is given by:

$$\exp\left(-(\omega/Y_R\dot{\epsilon})\rho L^2 e^{-\Delta E(\sigma)/kT} f(\Delta E(\sigma))d\Delta E d\sigma\right). \quad (4)$$

Thus, the probability that all defects (and hence the graphene sheet) survives the stress increment $d\sigma$ in time dt is obtained by integrating over the distribution of defects barrier heights, and is given by:

$$\exp\left(-(\omega/Y_R\dot{\epsilon})\rho L^2 \int_0^\infty e^{-\Delta E(\sigma)/kT} f(\Delta E)d\Delta E d\sigma\right). \quad (5)$$

Finally, the probability that the sheet survives till stress σ (time $t = \sigma/Y_R\dot{\epsilon}$) is again obtained by taking a product of the survival probabilities over small increments, which corresponds to integrating the term in the exponential in the above equation, and is given by:

$$S(\sigma) = \exp\left(-(\omega/Y_R\dot{\epsilon})\rho L^2 \int_0^\sigma \int_0^\infty e^{-\Delta E(\sigma')/kT} f(\Delta E)d\Delta E d\sigma'\right). \quad (6)$$

To make connection to the theory of extreme value statistics and Weibull distribution, note that the above can be written as:

$$S(\sigma) = \exp\left(-\omega Y_R \int_0^\sigma \int_0^\infty e^{-\Delta E(\sigma')/kT} f(\Delta E)d\Delta E d\sigma'\right)^{\rho L^2/\dot{\epsilon}}. \quad (7)$$

The right hand side of the above equation is of the form $F(\sigma)^{\rho L^2/\dot{\epsilon}}$, where $F(\sigma)$ can be identified with the exponential term. An important theorem from extreme value theory[8–10] tells us that for any distribution function $F(\sigma)$, the following holds under very mild restrictions:

$$F(\sigma)^N \to \Lambda((\sigma - b_N)/a_N), \quad (8)$$

where $\Lambda(\cdot)$ is of the Weibull, Gumbell or Frechet form, and a_N, b_N are constants. This is a very powerful result, in that it does not depend on the details of the distribution function $f(\Delta E)$; thus we do not need detailed knowledge of the distribution of defects to obtain a very good approximation of the global survival probability. The Weibull form emerges whenever the function $F(\sigma)$ has a power-law tail. For the Weibull form the function $\Lambda(\sigma)$ is given by $\exp(-\sigma^m)$, where m is a positive real number. Under this assumption (which we verify numerically), we can approximate the survival probability as:

$$S(\sigma) = F(\sigma)^{\rho L^2/\dot{\epsilon}} \to \exp\left(-\left(\frac{\sigma - b}{a}\right)^m\right), \tag{9}$$

where a, b are constants that depend on $\rho L^2/\dot{\epsilon}$.

The scaling suggested in Eq. 3 of the text can be arrived at on the basis of the physical reasoning hinted at in the manuscript; however, here we take a more mathematical approach. The assumption that $\Lambda(\cdot)$ is of the Weibull form essentially amounts to assuming that $F(\sigma)$ has a power law expansion, i.e.,

$$F(\sigma) \sim 1 - \alpha(\sigma - \sigma_0)^m + h.o.t, \tag{10}$$

then,

$$F((\alpha N)^{-1/m}\sigma + \sigma_0)^N \sim \left(1 - \frac{\sigma^m}{N}\right)^N \to e^{-\sigma^m}, \tag{11}$$

which gives:

$$F(\sigma)^N \to \exp\left(-\left(\frac{\sigma - \sigma_0}{(\alpha N)^{-1/m}}\right)^m\right) = \exp\left(-N\left(\frac{\sigma - \sigma_0}{(\alpha)^{-1/m}}\right)^m\right). \tag{12}$$

Thus,

$$S(\sigma) = F(\sigma)^{\rho L^2/\dot{\epsilon}} \to \exp\left(-\rho L^2 \dot{\epsilon}\left(\frac{\sigma - \sigma_0}{(\alpha)^{-1/m}}\right)^m\right). \tag{13}$$

Finally, realizing that the density of defects goes as $\rho \approx c/\mu^2$, where μ is the linear grain size, and c is a constant, and normalizing with a reference strain rate $\dot{\epsilon}_0$ gives us:

$$S(\sigma|L, \mu, \dot{\epsilon}) = \exp\left(-\frac{(L/\mu)^2}{\dot{\epsilon}/\dot{\epsilon}_0}\left(\frac{\sigma - \sigma_0}{v}\right)^m\right), \tag{14}$$

where $v = (\dot{\epsilon}_0/c\alpha)^{1/m}$.

Supplementary Note 3. Statistical methods

Maximum likelihood estimator for survival distribution of strength

Given the survival distribution function for the strength of nanocrystalline graphene $S(\sigma|L, \mu, \dot{\epsilon})$, the corresponding probability density is:

$$s(\sigma|L, \mu, \dot{\epsilon}) = -\partial_\sigma S(\sigma|L, \mu, \dot{\epsilon})$$
$$= \exp\left(-\frac{(L/\mu)^2}{\dot{\epsilon}/\dot{\epsilon}_0}\left(\frac{\sigma-\sigma_0}{v}\right)^m\right)\frac{(L/\mu)^2}{\dot{\epsilon}/\dot{\epsilon}_0}\frac{m}{v}\left(\frac{\sigma-\sigma_0}{v}\right)^{m-1}. \quad (15)$$

If for a single dataset the data σ_i are observed, then the corresponding log-likelihood function is given by $\sum_i \log s(\sigma_i|L, \mu, \dot{\epsilon})$. If we have q datasets $D_1,\ D_2, \ldots, D_q$ consisting of $n_1,\ n_2, \ldots, n_q$ data points, obtained at configurations $(L_1, \mu_1, \dot{\epsilon}_1),\ \ldots, (L_q, \mu_q, \dot{\epsilon}_q)$, then the parameters $\sigma_0,\ v,\ m$ can be obtained by a joint fit of data obtained by maximizing the following log likelihood function:

$$\mathcal{L} = \sum_{j=1}^{q}\sum_{i=1}^{n_j}\frac{1}{n_j}\log s(\sigma_i^j|L_j, \mu_j, \dot{\epsilon}_j), \quad (16)$$

where the dataset D_j consists of observations $\sigma_1^j,\ \sigma_2^j,\ \ldots,\ \sigma_{n_j}^j$ of fracture strengths. The normalization with the number of points in the dataset, n_j, is carried out to avoid a bias towards datasets with larger number of observations, because these are typically datasets with smaller values of L that can be simulated at smaller numerical cost.

Maximum likelihood estimator for survival distribution of toughness

The estimation of parameter α for the toughness model (Eq. 3 of the main text) is similar to the discussion in the previous section. However, one key difference should be noted. In the case of strength we were able to get a closed form formula and take the derivative in Supplementary Eq.15 analytically. As the corresponding operation cannot be performed analytically with Eq. 3 of the main text, the derivative has to be taken numerically by using finite differences.

Supplemantary References

[1] Ophus C., Shekhawat A., Rasool H., and Zettl A. Large-scale experimental and theoretical study of graphene grain boundary structures. *Phys. Rev. B*, 92:205402, Nov 2015.

[2] Plimpton S. Fast parallel algorithms for short-range molecular dynamics. *Journal of Computational Physics*, 117(1):1 – 19, 1995.

[3] Daivis P.J. and Todd B.D. A simple, direct derivation and proof of the validity of the sllod equations of motion for generalized homogeneous flows. *Journal of chemical physics*, 124(19):194103, 2006.

[4] Stuart S.J., Tutein A.B., and Harrison J.A. A reactive potential for hydrocarbons with intermolecular interactions. *Journal of Chemical Physics*, 112(14):6472–6486, 2000.

[5] Brenner D.W., Shenderova O.A., Harrison J.A., Stuart S.J., Ni B., and Sinnott S.B. A second-generation reactive empirical bond order (rebo) potential energy expression for hydrocarbons. *Journal of Physics: Condensed Matter*, 14(4):783, 2002.

[6] Grantab R., Shenoy V.B., and Ruoff R.S. Anomalous strength characteristics of tilt grain boundaries in graphene. *Science*, 330(6006):946–948, 2010.

[7] Yazyev O.V. and Louie S.G. Topological defects in graphene: Dislocations and grain boundaries. *Phys. Rev. B*, 81:195420, May 2010.

[8] De Haan L. and Ferreira A. *Extreme Value Theory: An Introduction.* Springer, 2007.

[9] Gumbel E.J. *Statistics of Extremes.* Columbia University Press, New York, 1958.

[10] Resnick S.I. *Extreme Values, Regular Variation and Point Processes.* Springer Verlag, New York, 2007.
